# Comparing the predictive value of plasma p-tau217 and CSF biomarkers (p-tau181, Aβ42) using amyloid positron emission tomography in Alzheimer’s continuum

**DOI:** 10.21203/rs.3.rs-7327653/v1

**Published:** 2025-09-26

**Authors:** Mohammad Hossein Azaraein, Elham Zarrazvand, Fatemeh Heydari, Nasim Asimi, Elham Ramezannezhad, Deniz Rahimipour, Kimia Janeshin, Aida Mahdian, Maryam Fallahpour Sichani, Shaghayegh Farhadi, Razieh Zamiri, Mohammad Amir Amirian, Maryam Bemanalizadeh

**Affiliations:** Shahid Sadoughi University of Medical Sciences and Health Services; Tehran University of Medical Sciences; Qom Islamic Azad University; Islamic Azad University, Tehran; Isfahan University of Medical Science; Iran University of Medical Sciences; Shahid Beheshti University of Medical Sciences; Kashan University of Medical Sciences; Islamic Azad University, Tehran; Islamic Azad University Sanandaj Branch; University of Padua; Kashan University of Medical Sciences and Health Services; NeuroTRACT Association, Tehran University of Medical Sciences

**Keywords:** Alzheimer’s Disease, Positron emission tomography, Phosphorylated-tau, Plasma biomarker, β-amyloid, cerebrospinal fluid biomarkers, cognitive impairment

## Abstract

**INTRODUCTION:**

This study explores the diagnostic accuracy of different biomarkers for Alzheimer’s disease (AD) using data from the Alzheimer’s Disease Neuroimaging Initiative (ADNI).

**METHODS:**

A cross-sectional analysis was performed on individuals with mild cognitive impairment. Key biomarkers assessed included plasma p-tau217, CSF p-tau181, CSF Aβ42, and amyloid PET imaging, alongside clinical assessments using the Clinical Dementia Rating (CDR) and the Alzheimer’s Disease Assessment Scale (ADAS).

**RESULTS:**

Plasma p-tau217 showed the strongest correlation with amyloid-β deposition and clinical scores (Adjusted R^2^ = 0.53 for ADAS, 0.51 for CDR), outperforming CSF Aβ42 and CSF p-tau181.

**DISCUSSION:**

These results suggest that plasma p-tau217 may serve as a more accurate, cost-effective, and non-invasive alternative to CSF biomarkers and PET imaging. Its superior predictive power highlights its potential in routine clinical settings for early diagnosis and monitoring of AD progression, addressing key challenges in accessibility and patient compliance associated with current diagnostic methods.

## 1. Introduction

Alzheimer’s disease (AD) is the most prevalent form of dementia, affecting approximately 50 million people worldwide, and this number is expected to triple by 2050, exacerbating the risks of disability, disease burden, and healthcare expenditure (1). AD is characterized by the abnormal accumulation of amyloid-β (Aβ) peptides and hyperphosphorylation of tau. Aβ deposition, occurring decades before the onset of clinical symptoms of AD, represents the earliest detectable pathological hallmark (2, 3).

The Aβ hypothesis posits that the accumulation of Aβ peptides, primarily Aβ40 and Aβ42, is the principal factor in AD pathogenesis. The deposition of Aβ peptides occurs mainly in the hippocampus, neocortex, and cerebrovascular regions, correlating with the distribution of senile plaques (4). Amyloid positron emission tomography (PET) imaging is an advanced non-invasive diagnostic method for AD is a utilizes radiopharmaceutical method performs by using several radiotracers such as 11C-Pittsburgh compound B and 18F-labeled compounds that selectively bind to Aβ plaques, evaluating Aβ plaque deposition in the brain’s grey matter, which is one of the earliest detectable changes in AD diagnosis (5, 6).

Recent advances in AD diagnosis have introduced biomarkers capable of identifying AD pathologies, similarly to how accurate Aβ and tau burdens are detected via PET or CSF analysis. It has been proven that biomarkers derived from plasma, urine, or cerebrospinal fluid (CSF) are pivotal in disease diagnosis, prognosis, and contribute significantly to the advancement of new drug therapies (7). For instance, CSF biomarkers such as total tau (t-tau), phosphorylated tau (p-tau), and the 42-amino acid isoform of Aβ (Aβ1–42) are favorable, as elevated t-tau, p-tau181, p-tau217 levels and reduced Aβ1–42 are observed in many diagnosed patients (8). Blood-based biomarkers provide less invasive and more affordable options compared to neuroimaging and CSF biomarkers, while due to the invasiveness of lumbar punctures, considerable costs, and the specialized facilities required for PET imaging, these approaches are restricted and lead to major worries for patients and limited accessibility (9, 10). It is noteworthy to mention that blood biomarkers are increasingly valuable for trial recruitment, diagnosis, and disease monitoring, potentially reducing the need for confirmatory CSF or PET scans (7, 11).

Moreover, because of minimal fold-change between amyloid PET-positive and -negative in patients, the reliability of the plasma Aβ42/40 ratio is inadequate for clinical investigations. In contrast, p-tau blood tests show a significant increase of 100–400% in AD patients, aligning with extracellular Aβ plaque pathology, which can be used as a defining hallmark of AD. This makes the p-tau one of the best contenders among novel AD blood tests, offering superior diagnostic accuracy and disease specificity to other known blood biomarkers. Therefore, p-tau, especially p-tau217, is recognized as the primary blood biomarker for AD pathology across all disease stages and exhibits high performance in distinguishing AD from other neurodegenerative diseases and detecting AD pathology in patients with mild cognitive impairment (MCI) (12–14).

This study aims to validate plasma p-tau217, specifically its accuracy as a predictive biomarker for Aβ burden in the early stages of Alzheimer’s disease. We hypothesize that plasma p-tau217 outperforms classic CSF biomarkers in predicting cerebral amyloid levels. Utilizing the Alzheimer’s Disease Neuroimaging Initiative (ADNI) database, we conducted a cross-sectional analysis comparing CSF biomarkers (p-tau181, Aβ42) with p-tau217 in predicting Aβ deposition in the brain.

## 2. Method

### 2.1. Participants

The data employed in this study were obtained from the Alzheimer’s Disease Neuroimaging Initiative (ADNI) database, which is available at (http://adni.loni.usc.edu). Launched in 2003, ADNI runs as a partnership between the public and private sectors under the guidance of Principal Investigator Michael W. Weiner, MD. The primary objective of ADNI has been to assess the PET and additional biological biomarkers alongside neuropsychological assessments to monitor the progression of MCI and early AD. The inclusion and exclusion criteria for ADNI participants are comprehensively detailed in other publications (15, 16). In summary, eligible individuals, aged 55 to 90, underwent various assessments, including neuroimaging, lumbar punctures, and longitudinal follow-ups. All methods were carried out in accordance with relevant guidelines and regulations.

For this investigation, we included participants classified as EMCI (early mild cognitive impairment) and LMCI (late mild cognitive impairment) from the ADNI cohort. We had access to all demographic information (such as gender, age, and education), CSF biomarkers (P-tau181 and Aβ42), plasma biomarker (p-tau217), and neuropsychological assessments, including the Alzheimer’s Disease Assessment Scale 11 (ADAS-11) and Clinical Dementia Rating (CDR). Additionally, we collected data from individuals who had undergone comprehensive amyloid PET data using [18F] florbetapir (FBP) smoothed to a uniform resolution of 6 mm. All datasets used for this study and the corresponding access dates are listed in Table S1. The study ultimately included a total of 30 participants, with 14 classified as EMCI and 16 as LMCI subjects.

### 2.2. Cognitive Assessments

Our study utilized two assessment methods, CDR (Clinical Dementia Rating) (17, 18) and ADAS-11 (Alzheimer’s Disease Assessment Scale) (19, 20), to evaluate the extent of cognitive and non-cognitive dysfunction in individuals exhibiting signs of the dementia spectrum. CDR is a scale that can determine the degree of dysfunction in six domains of cognitive and non-cognitive performance associated with dementia spectrum disorders, such as AD. These domains are memory, orientation, problem-solving, home and hobbies, community affairs, and personal care. Each domain is scored on a 4-point scale ranging from 0 to 3, where 0 indicates no impairment and 3 indicates severe impairment in the assessed function. The only exception is personal care, which is assessed on a 4-point scale without a 0.5 rating. The ratings are derived from a semi-structured interview conducted by a professional with the patient and reliable informants. The ADAS-11 consists of two parts: cognitive and non-cognitive testing. It was originally designed to measure the severity of cognitive and non-cognitive dysfunction in individuals with AD. The cognitive subscale, known as ADAS-Cog, is more commonly used and apart from assessing memory, praxis, language, and orientation, it encompasses specific tasks such as word recall and recognition, object and finger naming, and following commands. The assessment typically requires up to 45 minutes to complete, and the total score ranges from 0 to 70, with a higher score indicating greater cognitive impairment.

### 2.3. Plasma p-tau217 and CSF Biomarkers Measurements

Plasma p-tau217 was quantified in samples from the ADNI2/3 cohort using a standardized protocol developed with Janssen R&D. Measurements were performed on the Simoa HD-X platform with a three-step assay targeting p-tau217 and total tau, employing pT3 and hT43 antibodies specific to amino acids 210–220. The assay was calibrated with synthetic peptides (0.002–10 pg/mL) and used K2-EDTA plasma at a 1:2 dilution (linear up to 1:16). Validation confirmed high sensitivity, accuracy, and precision (intra-assay CV: 5.06 ± 4.09%; inter-assay CV: 4.16 ± 6.78%), along with sample stability across multiple conditions (21).

CSF biomarkers—Aβ42 and p-tau181—were measured to assess brain amyloid and tau pathology. Reduced Aβ levels indicate amyloid plaque accumulation, a hallmark of AD, while elevated p-tau181 reflect neurofibrillary tangles and neurodegeneration, respectively. Concentrations were determined using the INNO-BIA AlzBio3 assay (Fujirebio Europe), a Research Use Only (RUO) multiplex immunoassay on the Luminex platform, enabling simultaneous and precise quantification. The assay’s performance was validated with non-ADNI CSF samples, showing reliable within-laboratory precision but variable inter-laboratory reproducibility with ADNI1 CSF. A linear regression-based rescaling method was applied to mitigate kit-to-kit variability, using fresh ADNI baseline CSF aliquots to align subsequent batch results with original values, ensuring consistency and reproducibility (21).

### 2.4. Amyloid PET Measurements

Amyloid PET data were obtained using the Laboratory of Neuro Imaging (LONI) pipeline, incorporating tracers such as [18F] florbetapir (FBP). Amyloid burden was assessed in the cortical summary region, normalized by the whole cerebellum reference region. PET images were co-registered to compute regional Standardized Uptake Value Ratios (SUVRs). Amyloid positivity or negativity was determined using composite reference thresholds of 0.78 SUVR for FBP and 0.74 SUVR for FBB in 6-mm datasets

### 2.6. Statistical Analysis

Data were matched based on exam dates, visit codes, and variable values (e.g., plasma p-tau217, Aβ PET, CDR). Outliers were removed, and data were standardized using z-score normalization prior to analysis. Multiple linear regression analyses were conducted using Python (with pandas, NumPy, Matplotlib, and Seaborn packages). Six models were developed to assess the predictive value of plasma p-tau217, CSF p-tau181, and CSF Aβ42 for Aβ PET scores (SUVR/WC), with age, gender, and cognitive scores (CDR or ADAS) included as covariates. Each model evaluated one biomarker as the primary predictor, adjusting for the specified covariates, and results were compared across models to determine relative predictive performance.

## 3. Result

### 3.1. Data pre-processing

A cross-sectional study was conducted to identify individuals with complete records of plasma p-tau217, CSF p-tau181, CSF Aβ42, and summary SUVR normalized with the whole cerebellum (WC) by considering CDR and ADAS as cognitive scores. Among all the participants, thirty contributors met our necessary inclusion criteria for the study. Due to the clinical significance of degeneration changes, Evaluations were conducted at either 48 or 60 months, with the majority of patients visited at 48 months from 2016 to 2017.

### 3.2. Demographic characteristics of participants

We investigated patients containing 63% males and 37% females, diagnosed with either EMCI (47%) or LMCI (53%), with a mean age of 72 ± 6.4 years. Table 1 presents a summary of participants’ demographic data comprising CDR and ADAS scores, plasma p-tau217, CSF p-tau181, and CSF Aβ42 levels.

### 3.3. CDR-adjusted Models

After adjusting for age, gender and CDR, multiple linear regression analysis was conducted to evaluate the predictive value of biomarkers, considering SUVR/WC as the dependent criterion (Table 2). Our analysis demonstrated that our three biomarkers, including plasma p-tau217 (p < 0.001, Adj-R^2^=0.51), CSF p-tau181 (p < 0.001, Adj-R^2^=0.43), and CSF Aβ42 (p < 0.001, Adj-R^2^ =0.42), were significantly predictive for Aβ burden in the cognitively impaired Individuals.

### 3.4. ADAS-adjusted Models

In the following, by adjusting for age, gender and ADAS, multiple linear regression analysis was performed to assess the prediction rate of biomarkers’ effects on Aβ-pet scan score (Table 3). Our analyses showed that collected biomarkers were significantly predictive of Aβ deposition in the brain tissue [plasma p-tau217 (p < 0.001, Adj-R^2^=0.53), CSF Aβ42 (p < 0.001, Adj-R^2^=0.32), and CSF p-tau181 (p < 0.001, Adj-R^2^=0.37)].

### 3.5. Predictive Accuracy of Plasma Tau217, CSF P-tau181and CSF Aβ42 Biomarkers

By performing multiple linear regression analyses, after adjusting for demographic features, we tested the predictive ability of plasma p-tau217, CSF p-tau181, and CSF Aβ42 for assessing the accumulation of Aβ plaques using Aβ PET (SUVR/WC score). As we found, collected biomarkers had a remarkable relation with the Aβ deposition, showing significant predictive value. According to the analyses, plasma p-tau217 with a higher level of determination was the most powerful predictor in these analyses (Adj-R^2^=0.53 for ADAS and Adj-R^2^=0.51 for CDR).

As the figure shows, after adjusting for demographic features and cognition scores, Aβ deposition in the brain is meaningfully associated with the biomarkers. It indicates that an increase in plasma p-tau217 and CSF p-tau181 is correlated with enhancing the rate of Aβ burden in the brain tissue and CSF Aβ42 has an inverse concomitant with Aβ deposition.

## 4. Discussion

### 4.1. Interpretation

This study evaluated the predictive accuracy of three biomarkers, including plasma p-tau217, CSF p-tau181, and CSF Aβ42 and brain Aβ deposition measured by SUVR/WC in individuals with EMCI and LMCI. We evaluated each biomarker’s value in indicating brain Aβ pathology and their respective roles in diagnosing and the progression of AD. Our findings demonstrated a strong positive correlation between each of the biomarkers (plasma p-tau217, CSF p-tau181 and CSF Aβ42) and brain Aβ burden (p < 0.001), highlighting their predictive value in Aβ pathology. Meanwhile, plasma p-tau217 emerged as the strongest predictor of brain Aβ deposition, showing the highest Adj-R^2^ value after being adjusted for confounders, including age, gender, and cognitive scores (ADAS and CDR). These findings underscore plasma p-tau217’s potential as a reliable indicator of AD pathology in the early stages, as supported by multiple regression models.

As many studies have implied that CSF Aβ42 decreases during the development of AD, even preceding cognitive symptoms or Aβ-PET changes, its efficiency has been challenged by the research, demonstrating higher specificity for other biomarkers, such as tau proteins and neurofilament light chains in differentiating AD from other dementias (22–25). Moreover, CSF Aβ reduction can also occur in various neurodegenerative disorders, limiting its diagnostic precision, such as frontotemporal dementia, dementia with Lewy bodies, vascular dementia, Creutzfeldt-Jakob disease, and also it may also appear in individuals who never progress to AD clinical symptoms (26, 27). The adversity of CSF Aβ as a specific AD biomarker has led to increased interest in p-tau markers. Tau staging shows site-specific phosphorylation changes correlating with disease progression, structural, metabolic, neurodegenerative, and clinical disease indicators. The accumulation of p-tau217 and p-tau181 may begin concurrently with early Aβ aggregation, up to two decades before tau pathology becomes apparent (28).

Lai et al (2024) proved that plasma p-tau217 and p-tau181 have a great association with amyloid-PET status that can predict amyloid positivity. Besides, plasma epitopes of p-tau, such as 217 and 181, indicated a great relationship with their CSF levels, specifically for p-tau217. Due to the specificity and reliability of p-tau217 compared to other p-tau species found in previous studies, it can be used as the most suggestive predictor for diagnosis and progression analysis of AD (29), especially when plasma p-tau217 has the ability to predict long-term cognitive impairment in preclinical AD (30, 31).

Given these findings, plasma p-tau217 emerges as a clinically relevant biomarker. Unlike CSF biomarkers that require invasive lumbar punctures with associated risks such as infection and bleeding, or PET imaging, which are often impractical for routine use in primary care settings, plasma p-tau217 can be measured through a simple blood draw and lead to a reduction in the necessity for confirmatory testing. This minimally invasive approach significantly reduces patient discomfort while enabling more frequent and accessible monitoring of individuals at risk for AD (32, 33).

In the present study, the predictive value of CSF-Aβ42 and CSF p-tau181 were remarkable, as it was in line with previous studies (34, 35). Furthermore, we found that plasma p-tau217 biomarker exhibited greater results than CSF p-tau181 and CSF Aβ42 biomarkers, considering their significancy and higher Adj-R^2^ values for plasma p-tau217, which means plasma p-tau217 had stronger accuracy levels to be a stronger predictor of cognitive impairments and Aβ-PET positivity in the AD continuum. Our results were consistent with previous studies demonstrated that plasma p-tau217 accurately predicts abnormal Aβ status as well as CSF biomarkers in detecting Aβ burden in the brain and the upcoming onset of AD dementia in individuals with MCI (36–38). Likewise, in a meta-analysis, Qu et al (2021) confirmed the higher specificity of plasma p-tau217 in the diagnosis of AD compared to p-tau181 and p-tau231. It shows that plasma p-tau217 provided a superior indicator for AD detection and had better discriminative accuracy for MCI than p-tau181 and p-tau231 (38, 39).

### 4.2. Limitations and strengths

A relatively small sample size in our study may somewhat interfere with the generalizability of the results to a broader MCI population, particularly those outside the ADNI cohort. The cross-sectional design limits the ability to observe temporal changes in biomarker levels and disease progression over time, which would provide valuable insights into longitudinal dynamics. Additionally, our focus on MCI participants from the ADNI database may not capture the entire spectrum of cognitive decline associated with AD.

Despite these limitations, our study’s strengths significantly bolster the validity of the results and underscore its contributions to AD research. The integration of plasma p-tau217, CSF p-tau181, and CSF Aβ biomarkers offers a multifaceted perspective on amyloid pathology, enabling a more comprehensive understanding of AD-related changes, and the inclusion of advanced neuroimaging techniques, such as amyloid PET with standardized SUVR/WC metrics and CL scaling across multiple tracers, provides a gold-standard measure of amyloid deposition while ensuring consistency and comparability across analyses. Furthermore, employing dual cognitive assessments (CDR and ADAS) enhances both the reliability of cognitive evaluations and their associations with biomarker levels. These methodological strengths help address the study’s inherent limitations and support the conclusion that plasma p-tau217 demonstrates significant potential as a blood-based biomarker for evaluating amyloid deposition in individuals with MCI. In clinical practice, using blood-based biomarkers as a less-invasive and more cost-effective method could significantly impact disease assessment, especially in earlier stages, and streamline the process of patient follow-up. Moreover, the accuracy of plasma p-tau217 in diagnosing and predicting disease progression may complement conventional methods like CSF biomarkers and PET scans, potentially revolutionizing AD diagnostics and patient care.

### 4.3. Future direction

Comparing plasma and CSF biomarkers with other neuroimaging techniques, such as fluorodeoxyglucose-positron emission tomography scans (FDG-PET), can provide researchers more practical evidence to determine the accuracy of biomarkers’ prediction. Furthermore, combining plasma or CSF biomarkers with neuroimaging data can generate novel and more accurate methods of predicting AD.

Besides, conducting longitudinal studies and including more diverse cohorts would enhance understanding of plasma p-tau217’s predictive accuracy and utility in early stage AD diagnosis, future studies should track biomarker changes over timeframes of 3 to 6 years, as these periods have been commonly used in previous longitudinal research on AD biomarkers to monitor disease progression and transitions from preclinical to symptomatic stages (40). While its high predictive value signifies the potential clinical applications, further validation across various contexts, including disease progression monitoring and treatment response evaluation, should be granted. Furthermore, studies should consider the cost-effectiveness, ease of use, and integration into current diagnostic workflows.

## 5. Conclusion

Our findings indicated that predictive value of the plasma p-tau217 biomarker was stronger than CSF p-tau181 and CSF Aβ42. This suggests that p-tau217 is an accurate and non-invasive biomarker for early-stage AD diagnosis, pending further validation among various populations.

## Supplementary Material

Supplementary Files

This is a list of supplementary files associated with this preprint. Click to download.

- Supplementaryfile.docx

## Figures and Tables

**Figure 1:**
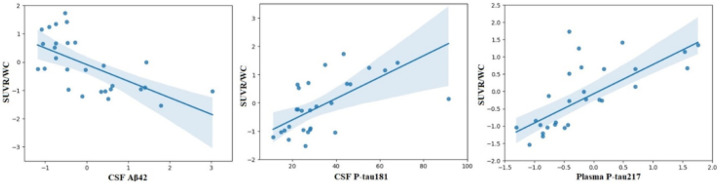
Scatter plot illustrating the correlations between biomarkers (plasma p-tau217, CSF p-tau181, and CSF Aβ42) and SUVR/WC scores, derived from multiple linear regression analyses.

**Table 1:** Demographics and clinical features

Variables	Mean ± SD/frequency
Age (years)	72 ± 6.4
Gender (Female, Male,%)	37%, 63%
EMCI, LMCI (%)	47%, 53%
CDR	1.59 ±2.2
ADAS-11	9.34 ± 7.8
plasma p-tau217	0.076 ± 0.05
CSF Aβ42	1113.6 ± 649
CSF p-tau181	33.2 ± 17.6
SUVR/WC	1.2 ± 0.23

Abbreviations: EMCI: early mild cognitive impairment; LMCI: Late Mild Cognitive Impairment; CDR: The Clinical Dementia Rating; ADAS: The Alzheimer’s Disease Assessment Scale–Cognitive Subscale; p-tau: phosphorylated tau; SUVR: Standardized Uptake Value Ratios; WC: whole cerebellum.

**Table 2:** Multiple Linear Regression Analysis using CDR

Predictor variables	Coeff	SE	P-value	Adj-R^2^
**Model 1**				
Plasma p-tau217	0.81	0.20	**< 0.001**	0.51
Age	0.09	0.14	0.51	
Gender	−0.19	0.28	0.49	
CDR	−0.05	0.08	0.51	
Intercept	0.19	0.24	0.45	
**Model 2**				
CSF p-tau181	0.03	0.01	**< 0.001**	0.42
Age	0.13	0.15	0.37	
Gender	−0.48	0.31	0.14	
CDR	0.12	0.06	0.05	
Intercept	−1.00	0.31	0.00	
**Model 3**				
CSF Aβ42	−0.50	0.15	**< 0.001**	0.43
Age	0.14	0.15	0.35	
Gender	−0.01	0.31	0.98	
CDR	0.12	0.06	0.05	
Intercept	−0.27	0.23	0.25	

Abbreviation: Coeff; Coefficient, SE; Standard error, Adj-R^2^; Adjusted R-squared, CSF; Cerebrospinal fluid, CDR; Clinical Dementia Rating.

**Table 3:** Multiple Linear Regression Analysis using ADAS

Predictor variables	Coeff	SE	P-value	Adj-R^2^
**Model 4**				
Plasma p-tau217	0.82	0.16	**< 0.001**	0.53
Age	0.07	0.13	0.61	
Gender	−0.23	0.28	0.42	
ADAS	−0.19	0.16	0.25	
Intercept	0.08	0.16	0.61	
**Model 5**				
CSF p-tau181	0.03	0.01	**< 0.001**	0.32
Age	0.17	0.16	0.30	
Gender	−0.60	0.35	0.09	
ADAS	0.03	0.18	0.87	
Intercept	−0.84	0.35	0.02	
**Model 6**				
CSF Aβ42	−0.56	0.16	**< 0.001**	0.37
Age	0.18	0.15	0.26	
Gender	0.01	0.33	0.98	
ADAS	0.20	0.15	0.20	
Intercept	−0.00	0.19	0.99	

Abbreviation: Coeff; Coefficient, SE; Standard error, Adj-R^2^; Adjusted R-squared, CSF; Cerebrospinal fluid, ADAS; Alzheimer’s Disease Assessment Scale.

## Data Availability

Data used in the preparation of this article were obtained from the Alzheimer’s Disease Neuroimaging Initiative (ADNI) database (http://adni.loni.usc.edu). The ADNI data are publicly available to qualified investigators upon application and approval. Further details on data access and availability can be found at the ADNI website. The dataset analyzed during this study is not publicly available due to privacy concerns. However, these data can be obtained from the corresponding author upon reasonable request.
